# Tracing a new path in the field of AI and robotics: mimicking human intelligence through chemistry. Part I: molecular and supramolecular chemistry

**DOI:** 10.3389/frobt.2023.1238492

**Published:** 2023-09-08

**Authors:** Pier Luigi Gentili, Pasquale Stano

**Affiliations:** ^1^ Department of Chemistry, Biology, and Biotechnology, Università degli Studi di Perugia, Perugia, Italy; ^2^ Department of Biological and Environmental Sciences and Technologies (DiSTeBA), University of Salento, Lecce, Italy

**Keywords:** chemical artificial intelligence, molecular probes, molecular motors, molecular machines, fuzzy molecules, molecular logic gates

## Abstract

Chemical Artificial Intelligence (CAI) is a brand-new research line that exploits molecular, supramolecular, and systems chemistry in *wetware* (i.e., in fluid solutions) to imitate some performances of human intelligence and promote unconventional robotics based on molecular assemblies, which act in the microscopic world, otherwise tough to be accessed by humans. It is undoubtedly worth spreading the news that AI researchers can rely on the help of chemists and biotechnologists to reach the ambitious goals of building intelligent systems from scratch. This article reports the first attempt at building a Chemical Artificial Intelligence knowledge map and describes the basic intelligent functions that can be implemented through molecular and supramolecular chemistry. Chemical Artificial Intelligence provides new tools and concepts to mimic human intelligence because it shares, with biological intelligence, the same principles and materials. It enables peculiar dynamics, possibly not accessible in software and hardware domains. Moreover, the development of Chemical Artificial Intelligence will contribute to a deeper understanding of the strict link between intelligence and life, which are two of the most remarkable emergent properties shown by the Complex Systems we call biological organisms.

## 1 Introduction

Humanity is experiencing a fourth technological revolution, altering how we live, work, and relate to one another (Schwab, 2016). Cutting-edge biotechnologies, nanotechnologies, Artificial Intelligence (AI), and Robotics are blurring the boundaries between biological, physical, and digital-virtual spaces. Specifically, AI and robotics assist us in our daily mental and manual efforts; they augment our intelligence through powerful computing machines (Wooldridge, 2022); they can even replace us in accomplishing specific tasks, sometimes going beyond human performances (Kurzweil, 2014). However, their functionalities are restricted to what they are programmed to do. In other words, AI and robots are still “weak” or “narrow” because they are capable of accomplishing specific tasks, but they cannot perform “general” intelligent actions as humans do (Goertzel, 2007; Russell and Norvig,2010). “General” AI will probably become a reality soon due to the hectic interdisciplinary research activities devoted to designing and constructing machines thinking humanely (Müller and Bostrom, 2016). The plethora of methods proposed to abstract human intelligence (Lehman et al., 2014) and develop AI might be grouped into two principal approaches. 1) The first approach relies on current general-purpose electronic computers or other special-purpose hardware and consists in writing software that can think rationally and humanly. Such software can reproduce the thinking process when it is a flow of rigorous logical operations (this strategy is known as the symbolic paradigm). Alternatively, software mimics some structural and/or functional features of neural networks to learn how to perform tasks from data (this latter strategy encompasses the subsymbolic and statistical paradigms) (Corea, 2019; Mitchell, 2019). 2) The second methodological approach to developing AI is reverse engineering of the brain in hardware, also known as neuromorphic engineering, which implements neural surrogates through non-biological systems. Such neural surrogates are used as neuroprostheses or to design brain-like computing machines that revolutionise von Neumann’s architectures of current electronic computers. Neural surrogates are implemented in hardware through specific solid materials. Such hardware is rigid if implemented through silicon-based circuits or inorganic memristors. It is flexible if based on organic semiconductor films (Nawrocki et al., 2016; Lee and Lee, 2019; Zhu et al., 2020; Christensen et al., 2022).

A brand-new idea for mimicking intelligence (intended in the broadest sense) has been recently sparked by three factors. Firstly, the relentless miniaturisation of the transistors (i.e., the basic computing elements of electronic computers), has been pushed to the limit, whereby a few atoms or single molecules become the basic switching elements (Fu et al., 2022). Secondly, it is clear that all living beings have the capacity to process physicochemical information through their bodies, which ultimately are fluid solutions. Thirdly, every living being, both pluricellular and unicellular, is provided with a sort of nervous system, consisting of sensors, effectors, and a sort of brain (Roederer, 2005), which are implemented through molecules and their networks. These three observations triggered the dawn and the initial development of the so-called Chemical Artificial Intelligence (CAI) (Gentili, 2013): a research line that exploits molecular, supramolecular, and systems chemistry in *wetware* (i.e., in fluid solutions) to imitate some performances of biological intelligence—at a minimal but organizationally significant level—and promote unconventional robotics, based on molecular assemblies.

This article is the first of two papers series, which aim to present our viewpoints on the current strategies for developing CAI, its paradigms, and its scope, also called the problems domain. Our opinions about its perspectives are also expressed. This first part focuses on Molecular and Supramolecular Chemistry. The second contribution will focus on Systems Chemistry.

It is undoubtedly worth spreading the news that AI researchers can rely on the help of chemists and biotechnologists to reach the ambitious goals of building intelligent systems from scratch. We expect that, in the following years, the collaboration among chemists, biotechnologists, computer scientists, mathematicians, engineers, and physicists, will boost the development of chemical AI and chemical robotics. From a technological viewpoint, CAI will contribute by providing new tools and concepts to the sciences of the artificial because it shares, with the intelligence of biological systems, the same principles and materials, enabling peculiar dynamics not accessible in software and hardware domains (or accessible via simulations, not by embodiment). Moreover, we suggest that CAI will also contribute to generating basic scientific knowledge by allowing a deeper understanding of the strict link between intelligence and life, which are two of the most astounding emergent properties shown by the Complex Systems we call biological organisms (Gentili P. L., 2021).

## 2 The field of CAI

Before presenting the strategies proposed so far to develop CAI and its domains of application, it is paramount to neatly outline its scope. First of all, the burgeoning research field of CAI must be distinguished from the already impactful and still tremendously soaring field of AI in chemistry (Baum et al., 2021), in which the usual chemists’ actions of design, synthesis, characterisation and application of molecules and materials are assisted and accelerated by the AI tools and methods (Butler et al., 2018) such as machine learning, quantitative structure-activity relationship (QSAR) models, molecular docking, chemical reaction prediction, generative models, chemoinformatics and data mining. Secondly, CAI refers to designing and implementing intelligent complex molecular systems, which—as it happened with the conventional AI path—have the human nervous systems and its components, i.e., the Sensory System (SS), the Central Nervous System (CNS), and the Effector System (ES) (ultimately made of single neural cells) as epitomes.

There is more, however. The features of CAI allow starting new investigation directions, in particular those inspired to lower and more basic, but nevertheless relevant, forms of intelligence, e.g., the one referred to uni- or pauci-cellular organisms. The biologically-inspired molecular systems of CAI are complex fluid mixtures, usually made of soft matter, likewise living cells. As it happens in living cells, the molecular elements literally transform into each other, blurring the difference between the “computer” and “computed” elements of the circuit. The result of these dynamics is a sort of computation that can be interpreted in the classical manner (input/output) or according to systemic perspectives: Indeed, these systems can display linear and circular causalities, and all perform parallel computations.

**FIGURE 1:**
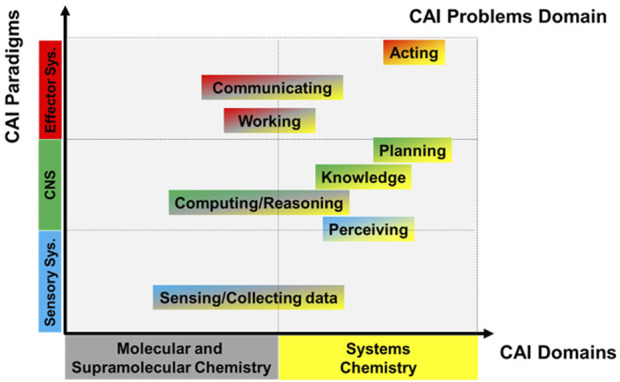
The Chemical Artificial Intelligence (CAI) knowledge map.

Figure 1 shows the first attempt at building a CAI knowledge map in analogy to that built for AI (Corea, 2019). It is a graph whose *x*-axis reports the CAI domains that are split, for convenience, into two parts: Molecular and Supramolecular Chemistry and Systems Chemistry, respectively. The *y*-axis reports the CAI paradigms, which are the main components of the Human Nervous System (HNS), i.e., the Sensory System (SS), the Central Nervous System (CNS), and the Effector System (ES). We use these terms here because of their importance in human-centered descriptions of intelligence, but it is clear that functionally similar parts or modules can also be found in other organisms, down to unicellular ones. The problems CAI faces are properly distributed between the two coordinates of the graph. The SS, which includes the sensory cells catching physical and chemical stimuli, allows for collecting data. The ability to transform raw sensorial inputs into usable information is perception and requires the brain (included in the CNS). The brain also allows computing and reasoning, i.e., solving problems. Knowledge is generated by the ability to understand and represent the world. Planning is the capability of fixing goals. The ES, made of muscles and glands, allows working, communicating, and acting, which means pursuing and achieving the planned goals to maximize the expected utility. The principal results achieved in mimicking human intelligence through Molecular and Supramolecular chemistry are highlighted in this manuscript, along with their perspectives. The results and perspectives related to Systems Chemistry will be presented in the second part of this series.

## 3 Molecular and supramolecular chemistry

**FIGURE 2:**
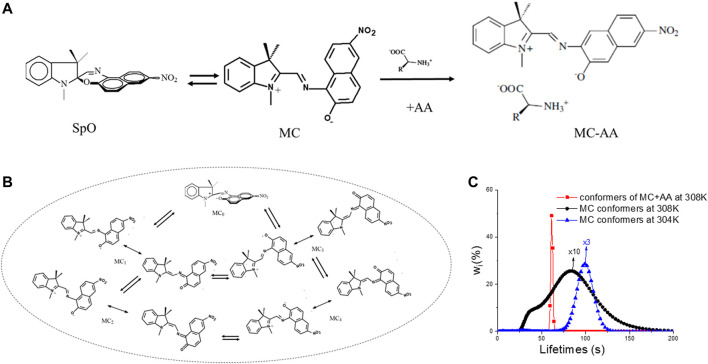
Two molecules (labelled as SpO and MC, respectively) with the same Molecular Formula but different structures are shown, along with a supramolecule generated by the interplay between an amino acid (AA) and MC in **(A)**. **(B)** Graph B displays a collection of conformations for MC. **(C)** Plot C depicts the lifetimes’ distribution for the MC conformers at 304 K (blue trace) and 308 K (black trace), and in the presence of AA at 308 K (red trace): the conformational distribution for MC gives rise to a molecular fuzzy set whose shape and position are clearly context-dependent.

The behavior of molecules and how they can be employed to develop CAI depend primarily on their structure. The molecular structure is defined by specifying i) the type and number of the atomic elements that are present (through the so-called “Molecular Formula”) ii) how the constitutive atoms are reciprocally bound (through the so-called “Molecular Structure”), and iii) how the groups of atoms are arranged in the three-dimensional space. Figure 2A shows an example of a molecule that can exist under two structures (labelled as SpO and MC) that have the same Molecular Formula ( $C_{21}H_{19}N_{3}O_{3}$ ) but they differ in how the atoms are bound. When two or a few more molecules establish sufficiently reciprocal strong links, they give rise to a supramolecular entity with emergent properties (Lehn, 1993). Figure 2A shows an example of a supramolecule generated by the electrostatic interaction between MC and an amino acid AA. Their interplay affects the property of both MC and AA.

### 3.1 Sensing

Most of the molecules and supramolecules respond to chemical and physical inputs by changing their structures. For any solution containing a number of responsive molecules of the order of Avogadro’s number (i.e., 10^23^), the outputs are macroscopic variables that can be monitored through proper instruments, which often are spectroscopic. Every output is a function of the inputs, the molecular structure, and the features of the surrounding microscopic environment. Such chemical compounds are sensors of the molecular world (De Silva, 2013). Since the molecular structures and properties can change in combinational (devoid of memory effects) or sequential (including memory) manners, the behaviour of these sensors can be exploited to process information (Credi, 2007; Szaciłowski, 2008).

### 3.2 Computing and communicating

When the molecules are maintained in coherent quantum states (through demanding procedures of isolations), they are employed to implement quantum computing, which is alluring for its parallelism (Bennett and Di Vincenzo, 2000; Nielsen and Chuang, 2004). When the random Brownian motion, sustained by the available thermal energy, induces the collapse of quantum states through molecular collisions, it is still possible to use molecules to compute (Gentili, 2011). When the molecular states available are two or a few more, the system can be used to process binary or multi-valued discrete logics. Since the early 1990s, molecules and supramolecules have been proposed as alternatives to solid inorganic semiconductors for implementing Boolean logic gates and functions (De Silva, 2013). Since it is possible to exploit several (spectroscopic) techniques to probe the input-output relationships, molecular logic gates are reconfigurable: the logic function depends on which output is monitored. It is challenging to connect different molecular logic gates to obtain extended circuits, as in electronics, due to the variety of physicochemical inputs and outputs that are usually involved. Therefore, molecular logic gates are inappropriate for implementing the “wet” counterparts of modern electronic computers, i.e., the so-called chemical or molecular computers. However, molecular and supramolecular logic gates remain valuable probes of the microscopic world (Pischel et al., 2013). They can reciprocally communicate through collisions, after diffusing closely, or through much swifter optical signals.

When the accessible molecular states are almost infinite, it is reasonable to exploit them for processing infinite-valued logic, such as fuzzy logic. Any chemical compound that exists under a plethora of conformers (i.e., potentially an infinite number of structures that differ in the 3D arrangement of the atoms) or is embedded in micro-heterogeneous environments (Gentili and Perez-Mercader, 2022) can be used to implement a fuzzy set. Figure 2B shows some conformers of MC: they are labelled as MC_0_, MC_1_, MC_2_, MC_3_, and MC_4_. MC_0_ is obtained as soon as the C-O bond of the spiro-oxazine (SpO) is broken. It is a sort of matrix of the other conformers that are in chemical equilibrium (represented by two opposite arrows) and are obtained by mutually rotating the two-halves of the molecule. Each conformer exists as a superposition (represented by the dual arrow) of two or more structures that differ in how the electrons are distributed across the molecular skeleton. The features of MC conformational distribution, which are the number of conformers and their relative weights (i.e., the $w_{i}$ values appearing in Figure 2C), depend on the microscopic physicochemical environment (Gentili, 2014). Figure 2C shows the conformational distributions of MC in three distinct conditions, knowing that different MC conformers have distinct lifetimes. The black and blue traces represent the situations at 308 K and 304 K, respectively: the lower the temperature, the narrower the conformational distribution. The red trace shows the remarkable effect played by the aminoacid AA that interplays with MC and reduces the number of MC conformers. Such conformational distributions become words of a chemical language (Gentili P. L., 2021), whose meaning is context-dependent, likewise to the words of any human language.

### 3.3 Working

Many of the molecules employed to compute respond to the stimuli by changing their skeleton through relative movements of some of its molecular fragments, which can be assimilated to the movement of parts of a macroscopic machine. In this case, the molecules are also called Molecular Machines. Some of them carry out useful work within a cycle, and they are named Motors. Some others are simple switches because they go back and forth between two states (Aprahamian, 2020). Molecular Machines are forced to work in that peculiar environment, which is the molecular world. It is dominated by the Brownian motion, fueled by thermal energy, and it is characterised by low Reynolds numbers. Any useful work must fight against the random motion of the particles and the viscous force exerted by the surrounding micro-environment (Bustamante et al., 2001). Molecular Machines perform work at the Ångstrom and nano-levels. Their work can be transferred to higher spatial scales (from the micro-to the macro-level) if the molecules are aligned in space and act synchronously. Alternatively, work can be hierarchically transferred from the nano-to the macro-level through Systems Chemistry, as explained in the mentioned second part of this series.

## 4 Conclusive remarks

Molecular and supramolecular chemistry is clearly contributing to the development of CAI. Responsive molecules and supramolecules are valuable for mimicking basic functions of the human nervous system and any other biological system, such as sensing, computing, communicating, and working. The chemical compounds that play these roles assist and augment humans because they establish a direct link between our macroscopic world and the microscopic molecular world. Among all the possible physicochemical variables that can help to link the two worlds, the optical ones are particularly appealing (Gentili, 2011). As inputs, they can be focused on tiny areas, selecting the number of molecules to communicate with. As outputs, they can be easily caught even by human eyes (if they have frequencies belonging to the visible region) or conveyed over long distances through optical fibres, which work as communication channels. Each chemical compound employed in CAI can accomplish just one or a limited range of tasks. In other words, molecules and supramolecules can promote “weak” forms of CAI. We might think of approaching “general” forms of CAI by assembling many types of molecules, each playing a peculiar function and giving rise to an autonomous chemical system capable of perceiving, planning, and acting (see Figure 1). Reaching “general” CAI will probably mean having the capability of controlling the transition from non-living to living matter, as will be commented on in our next manuscript.

## Data Availability

The original contributions presented in the study are included in the article/supplementary material, further inquiries can be directed to the corresponding author.
